# Correction: MicroRNA-379 inhibits the proliferation, migration and invasion of human osteosarcoma cells by targeting EIF4G2

**DOI:** 10.1042/BSR-20160542_COR

**Published:** 2020-07-02

**Keywords:** EIF4G2, Invasion, MicroRNA-379, Migration, Osteosarcoma, Proliferation

The authors of the original article “MicroRNA-379 inhibits the proliferation, migration and invasion of human osteosarcoma cells by targeting EIF4G2” (*Bioscience Reports* (2017) **37**, DOI: 10.1042/BSR20160542) would like to correct Figure 5, as they had mistakenly used images of from another group in place of the blank group in Figure 5F and H due to nonstandard naming of the images. The authors express their sincere apologies for any inconvenience that this error has caused to the readers. The corrected version of Figure 5 is presented here.

**Figure 5:**
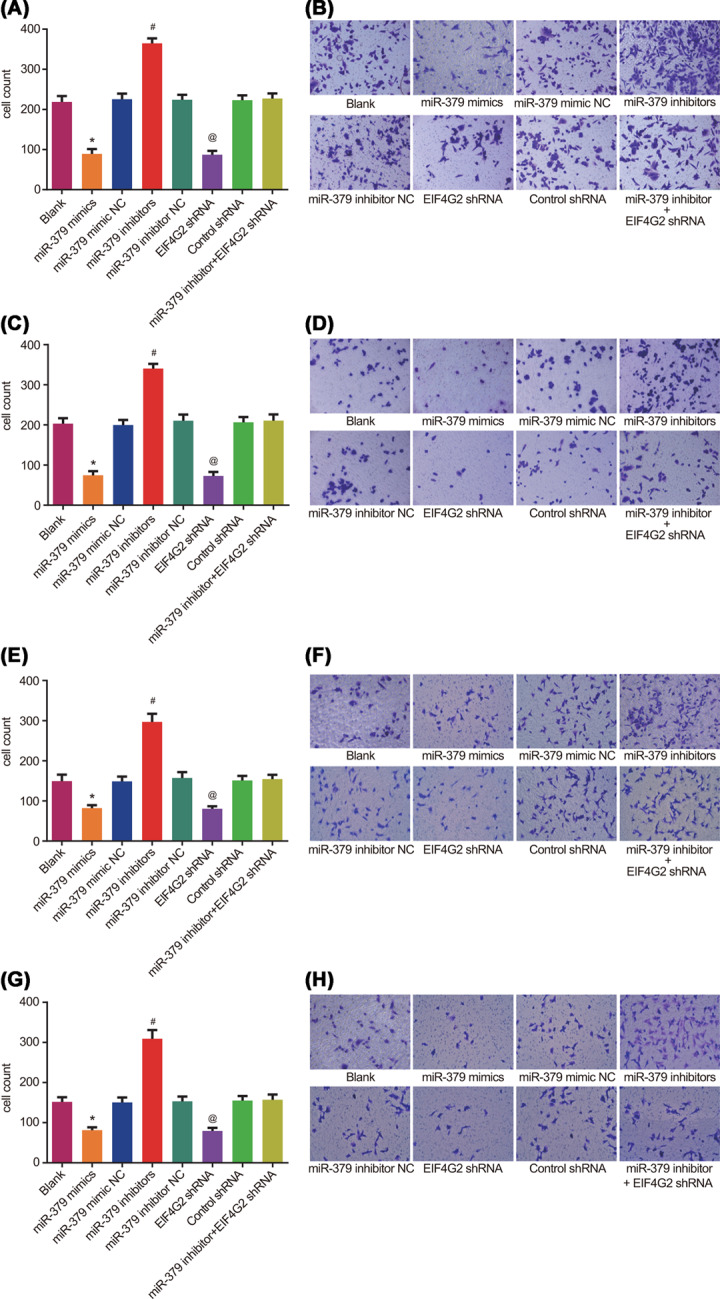
The migration and invasion abilities of U2OS and MG-63 cell lines detected by the Transwell assay (**A**) The number of invasive U2OS cells in each group; (**B**) images of the invasion of U2OS cells under a light microscope; (**C**) the number of invasive MG-63 cells in each group; (**D**) images of the invasion of the MG-63 cells under a light microscope; (**E**) the number of migrating U2OS cells in each group; (**F**) images of the migration of U2OS cells under a light microscope; (**G**) the number of migrating MG-63 cells in each group; (**H**) images of the migration of the MG-63 cells under a light microscope; *n* = 3; ^#^, *P*<0.05 compared with the *miR-379* inhibitor NC group; *, *P*<0.05 compared with the *miR-379* mimic NC group; @, *P* < 0.05 compared with the control shRNA group.

